# Targeted Delivery of Mesenchymal Stem Cell-Derived Nanovesicles for Spinal Cord Injury Treatment

**DOI:** 10.3390/ijms21114185

**Published:** 2020-06-11

**Authors:** Ju-Ro Lee, Jae Won Kyung, Hemant Kumar, Sung Pil Kwon, Seuk Young Song, In-Bo Han, Byung-Soo Kim

**Affiliations:** 1 School of Chemical and Biological Engineering, Seoul National University, Seoul 08826, Korea; ljr0518@snu.ac.kr (J.-R.L.); skccdr@snu.ac.kr (S.P.K.); sysong@snu.ac.kr (S.Y.S.); 2 Department of Neurosurgery, CHA university, CHA Bundang Medical Center, Seongnam-si 13448, Korea; kyungjaewon88@gmail.com; 3 Department of Pharmacology and Toxicology, National Institute of Pharmaceutical Education and Research-Ahmedabad, Gujarat 382010, India; hemantbhave@gmail.com; 4 Institute of Chemical Processes, Seoul National University, Seoul 08826, Korea; 5 Institute of Engineering Research, Seoul National University, Seoul 08826, Korea

**Keywords:** exosome-mimetic extracellular nanovesicles, macrophages, mesenchymal stem cells, spinal cord injury

## Abstract

Due to the safety issues and poor engraftment of mesenchymal stem cell (MSC) implantation, MSC-derived exosomes have been spotlighted as an alternative therapy for spinal cord injury (SCI). However, insufficient productivity of exosomes limits their therapeutic potential for clinical application. Moreover, low targeting ability of unmodified exosomes is a critical obstacle for their further applications as a therapeutic agent. In the present study, we fabricated macrophage membrane-fused exosome-mimetic nanovesicles (MF-NVs) from macrophage membrane-fused umbilical cord blood-derived MSCs (MF-MSCs) and confirmed their therapeutic potential in a clinically relevant mouse SCI model (controlled mechanical compression injury model). MF-NVs contained larger quantity of ischemic region-targeting molecules compared to normal MSC-derived nanovesicles (N-NVs). The targeting molecules in MF-NVs, which were derived from macrophage membranes, increased the accumulation of MF-NVs in the injured spinal cord after the in vivo systemic injection. Increased accumulation of MF-NVs attenuated apoptosis and inflammation, prevented axonal loss, enhanced blood vessel formation, decreased fibrosis, and consequently, improved spinal cord function. Synthetically, we developed targeting efficiency-potentiated exosome-mimetic nanovesicles and present their possibility of clinical application for SCI.

## 1. Introduction

Traumatic spinal cord injury (SCI) is a serious medical condition that causes permanent motor and sensory dysfunction. SCI is a complex dynamic process that initiates a complex cascade of secondary injury mechanisms including hemorrhage, ischemia, apoptosis, inflammation, demyelination, and glial scar formation [1]. The inhibitory environment around the axons suppresses the axonal regeneration, and the synaptic plasticity is insufficient for substantial functional improvement. Recently, mesenchymal stem cell (MSC) therapies have emerged for spinal cord repair because of its multifunctional therapeutic abilities for neuroprotection, anti-inflammation, and angiogenesis [2,3,4,5,6,7,8,9,10].

However, MSC therapies are limited by several obstacles. Direct injection of MSCs into the injured spinal cord could provide further damage [11]. In addition, systemically injected MSCs can rarely reach the lesion site because of the pulmonary entrapment of MSCs [11]. Moreover, the therapeutic efficacy of implanted MSCs can be limited because of the poor survival and the possibility of the differentiation into other cell types including chondrocytes and osteoblasts [12]. To avoid these limitations, MSC-conditioned medium has been suggested as an alternative to the transplantation of MSCs for SCI [13]. Recent studies have revealed that the therapeutic effects of MSCs are mainly attributed to exosomes, which are a type of extracellular vesicles that contain RNAs and proteins and serve as a nanocarrier of the biomolecules between cells [14,15]. Proteins and RNAs in exosomes are transferred to recipient cells and affect the recipient cells via protein functions and RNA translation [16,17]. In preclinical studies, MSC-derived exosomes have shown therapeutic potential for numerous diseases including myocardial infarction, ischemic vascular diseases, neurodegenerative diseases, and SCI [18,19,20,21,22,23,24]. MSC-derived exosomes exert therapeutic effects via anti-apoptosis, immunomodulation, anti-inflammation, and angiogenesis, which is similar to the therapeutic effects of MSCs. MSC-derived exosomes have a lower possibility of an immune response [25]. Importantly, MSC-derived exosomes therapies can avoid the major limitations of MSC therapy. MSC-derived exosome therapies can avoid the problems of poor MSC survival and possible MSC-differentiation into other cell types. Exosomes exhibit less entrapment in the lung following systemic administration due to their small size and can also be stored at −80 °C, which costs much less than MSC storage at −196 °C. In this context, MSC-derived exosome therapies have emerged as a substitute for MSC therapies [26].

Although exosomes therapies have shown therapeutic potential, large-scale production of exosomes is a barrier for clinical applications. One million MSCs secrete 1–4 μg of exosomes per day, which can hardly meet the demand for clinical trials [27,28]. Therefore, to produce sufficient amounts of exosomes from MSCs for clinical applications, cultures of a large number of MSCs and long-term cultures are required. To overcome these obstacles, recent studies have developed exosome-mimetic nanovesicles (NVs). NVs have particle size and component including intracellular RNAs, proteins, and plasma membrane proteins similar to those of exosomes and can transfer biomolecules from parent cells to recipient cells like exosomes [29,30]. NVs are generated through the serial extrusion of cells via microporous and nanoporous filters, while exosomes are secreted from cells. Importantly, 250-fold larger quantity of NVs can be obtained than exosomes from the same number of cells [31]. Furthermore, each NV contains a two-fold greater quantity of RNAs and proteins than each exosome [31]. Taken together, NVs would be advantageous over exosomes in terms of production, cost-effectiveness, and therapeutic efficacy. Therefore, in the present study, we used MSC-derived NVs for SCI treatment rather than MSC-derived exosomes.

**Figure 1:**
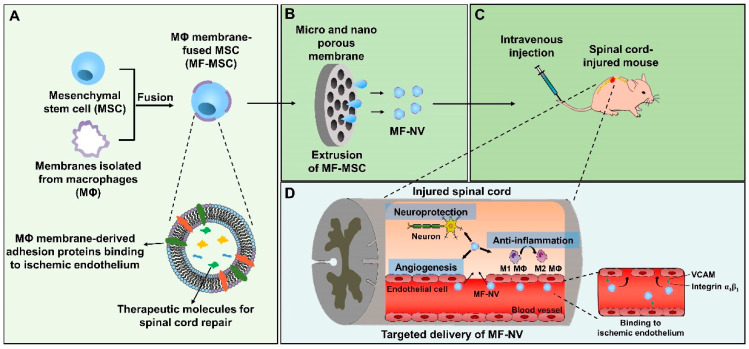
Schematic diagrams for the fabrication and therapeutic effects of macrophage membrane-fused exosome-mimetic nanovesicles (MF-NVs) for spinal cord repair. (**A**) The preparation of MF-mesenchymal stem cells (MSC) through the fusion of macrophage (MΦ) membranes into MSCs. MF-MSCs contain the membrane proteins of MΦ and the therapeutic molecules. (**B**) Production of MF-NVs from MF-MSCs by serial extrusion. (**C**) Intravenous injection of MF-NVs into the spinal cord-injured mouse. (**D**) The mechanisms of targeting and therapeutic effects of MF-NVs on spinal cord injury (SCI) mice model.

Despite the advantages of NVs and exosomes over MSCs therapies, NVs and exosomes do not have the ability to target to diseased organs after systemic injection, which limits the therapeutic efficacy of NV or exosome therapies [32,33]. To improve the diseased organ-targeting efficiency of NVs, here we fabricated macrophage membrane-fused MSCs (MF-MSCs) and then produced macrophage membrane-fused MSC-derived NVs (MF-NVs) through the extrusion of MF-MSCs **(Figure 1**). Macrophage membranes contain binding molecules such as very late antigen 4 (VLA4) comprising integrin α4 and β1 and the various receptors of inflammatory cytokines. The membrane proteins included in MF-NVs may facilitate the targeting of MF-NVs at the inflammatory tissues and scavenge inflammatory cytokines [34,35,36]. MF-NVs contain integrin α4 and β1 originated from macrophage membrane and may improve the efficiency of NVs targeting to ischemic and inflammatory organs such as the injured spinal cord. Here, we found that both normal MSC-derived NVs (N-NVs) and MF-NVs exhibited neuroprotective, anti-inflammatory, and angiogenic effects in vitro, which are the major therapeutic mechanisms of spinal cord repair. Following the systemic injection into mice with SCI, MF-NVs showed better spinal cord targeting efficiency and therapeutic efficacy than N-NVs.

## 2. Results

### 2.1. Characterization of MF-MSCs and MF-NVs

**Figure 2:**
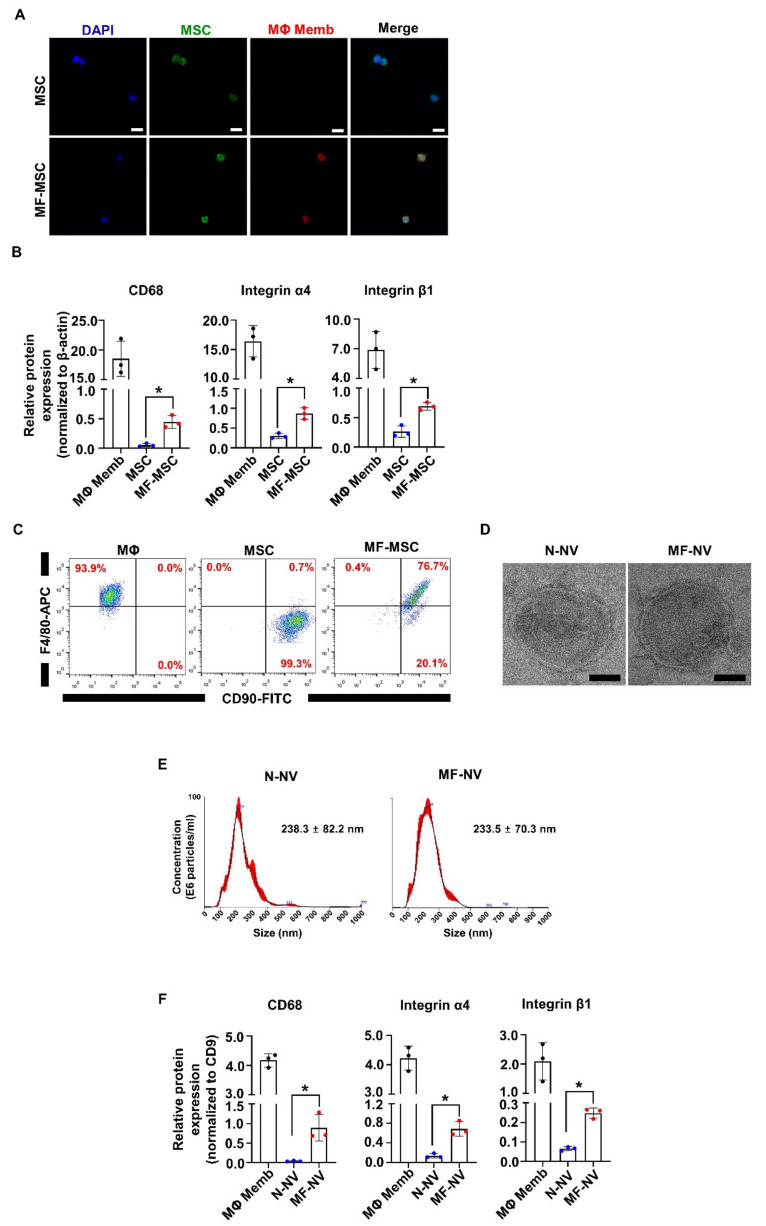
Characterization of MF-MSC and MF-NV. (**A**) Fluorescent confocal images of MSC and MF-MSC after fusion. Scale bars, 25 μm. (**B**) The quantification of Western blots of surface markers of MΦ membrane (CD68, integrin α4, and β1), MSC, and MF-MSC (n = 3 per samples, 20 μg of total proteins). (**C**) Flow cytometric analysis of MSC (CD90) and MΦ (F4/80) surface marker expressions on MF-MSC (n = 4 per samples). (**D**) Transmission electron microscopy of normal MSC-derived nanovesicles (N-NV) and MF-NV. Scale bars, 50 nm. (**E**) Size distribution of N-NV and MF-NV, as evaluated by nanoparticle tracking analysis (n = 4 per samples). (**F**) The quantification of Western blots of surface marker of MΦ membrane, N-NV, and MF-NV (n = 3 per samples, 20 μg of total proteins).

We first obtained the membranes of macrophages through dissociation and extrusion. The size distribution of macrophage membranes is shown in Figure S1. We then fused the membranes of macrophages into MSCs using polyethylene glycol (PEG). To determine whether the membranes were integrated into MSCs, we characterized the macrophage membrane-fused MSCs (MF-MSCs). Fluorescent microscopic images showed that DiO-labeled MSCs (green) were decorated with DiI-labeled macrophage membranes (red) (Figure 2A). To demonstrate that the overlapping of fluorescent signals was not due to dye transfer, we evaluated the surface markers of MSCs and MF-MSCs using Western blot analysis (Figure 2B and Figure S2). MF-MSCs contained CD68, a surface marker of macrophages, indicating that macrophage membranes were integrated into MSCs. Integrin α4 and integrin β1, known as VLA4, were also highly expressed in MF-MSCs than MSCs. VLA4 on the surface of circulating monocytes facilitates monocyte binding to the inflammatory endothelium [37]. The enhanced expression of VLA4 on the membrane of MF-MSCs indicated that MF-MSCs inherit the binding ability of macrophages to the inflammatory sites. Flow cytometry analyses confirmed that a macrophage marker (F4/80) was expressed on MF-MSCs, but not on MSCs (Figure 2C). An MSC marker (CD90) was expressed on both MSCs and MF-MSCs. Next, we prepared N-NVs and MF-NVs by serial extrusion of MSCs and MF-MSCs, respectively, through microporous and nanoporous membranes (Figure S3). TEM images revealed that both N-NVs and MF-NVs had spherical shapes (Figure 2D). The size distribution of N-NVs and MF-NVs was determined using nanoparticle tracking analysis (Figure 2E). N-NVs and MF-NVs showed mean sizes of 238.3 ± 82.2 nm and 233.5 ± 70.3 nm, respectively. The size distributions of NVs were similar to those of NVs in previous studies that exhibited therapeutic effects in vivo [30,38,39]. Western blot analysis revealed that both N-NVs and MF-NVs contained CD9, a marker for exosomes and NV (Figure 2F). The protein levels of CD68, integrin α4, and integrin β1, which are markers of macrophages, were significantly higher in MF-NVs than in N-NVs (Figure S4).

### 2.2. Anti-Apoptotic, Anti-Inflammatory, and Angiogenic Effect of MF-NV In Vitro

**Figure 3:**
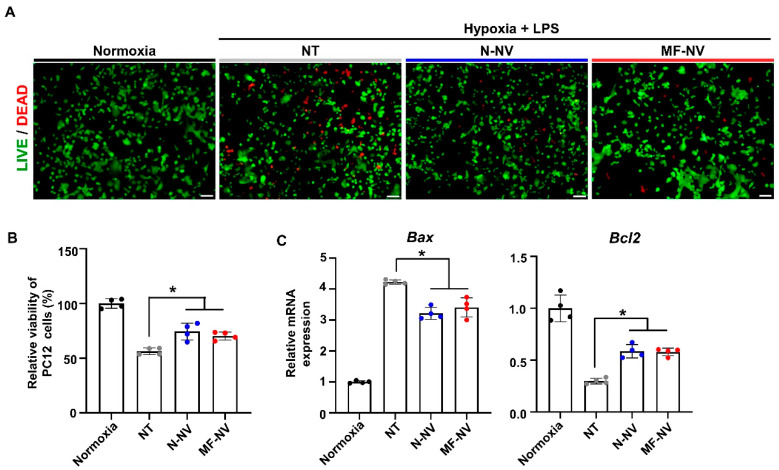
In vitro neuroprotective effects of NVs. (**A**) Representative images of viable and nonviable PC12 cells stained by fluorescein diacetate (FDA)/ethidium bromide (EB) after treatment. Scale bars, 100 μm. (**B**) Relative cell viability as evaluated by the CCK assay after the treatment (n = 4 per group). (**C**) Relative mRNA expression levels of apoptotic (*Bax*) and anti-apoptotic (*Bcl2*) genes in PC12 cells after treatment, as evaluated by qRT-PCR (n = 4 per group). NT indicates no treatment. * *p* < 0.05 by using one-way ANOVA followed by post-hoc Bonferroni test. All values are mean ± SD.

**Figure 4:**
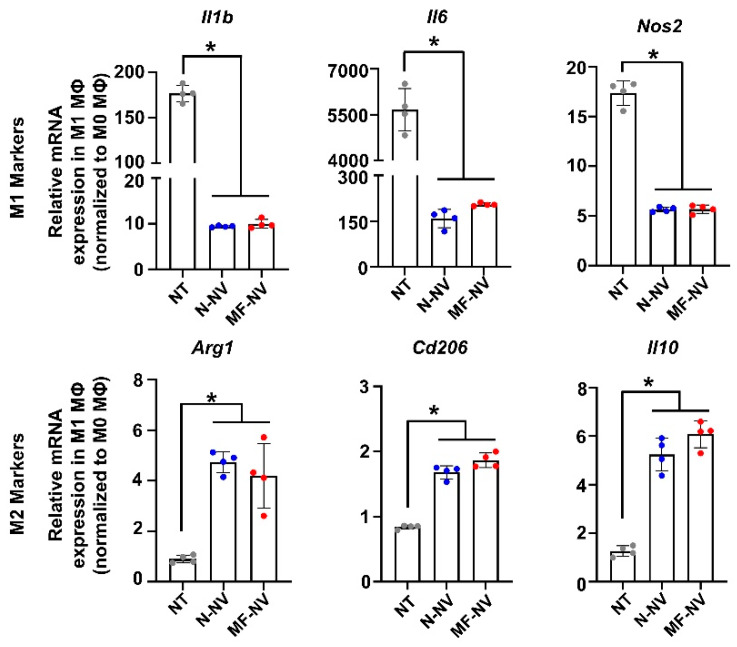
In vitro anti-inflammatory effects of NVs. Macrophage polarization after treatment. Relative mRNA expression levels in M1 MΦ of the markers of inflammatory M1 macrophages (*Il1b*, *Il6*, and *Nos2*) and reparative M2 macrophages (*Arg1*, *Cd206*, and *Il10*), as evaluated by qRT-PCR (n = 4 per group). All data were normalized to those of M0 MΦ. NT indicates no treatment. *p < 0.05 by using one-way ANOVA followed by post-hoc Bonferroni test. All values are mean ± SD.

**Figure 5:**
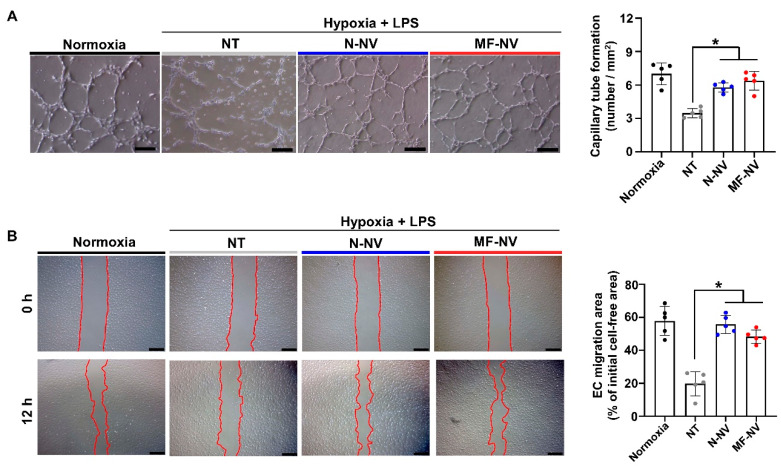
In vitro angiogenic effects of NVs. Representative images and the quantification data of (**A**) capillary tube formation and (**B**) cell migration of human umbilical vascular endothelial cells (HUVECs) after treatment. Red lines indicate borders of the cell-free area. Scale bars, 100 and 500 μm, respectively. NT indicates no treatment. * *p* < 0.05 by using one-way ANOVA followed by post-hoc Bonferroni test. All values are mean ± SD.

Next, we investigated in vitro effects of NVs on various types of cells (PC12 cell, RAW 264.7 cell, and human umbilical vascular endothelial cell (HUVEC)) that can reflect the cell types in spinal cord tissue. Previous studies have reported that exosomes from MSCs show neuroprotective effects, induce the polarization of macrophages from inflammatory state (M1) to reparative state (M2), and enhance angiogenesis by endothelial cells migration and proliferation [40,41,42]. First, to evaluate NV uptake by the cells in vitro, we treated fluorescently labeled N-NVs and MF-NVs to various types of cells for 24 h (Figure S5). The fluorescent images showed that N-NVs and MF-NVs were effectively internalized into the cells. We then examined the neuroprotective effects of NVs on neuron-like PC12 cells. PC12 cells were cultured under the ischemic and inflammatory conditions for 24 h, because SCI lesions show acute ischemia and inflammation that lead to apoptosis of neurons. Importantly, the treatment with NVs improved the survival of PC12 cells, as evaluated by live/dead staining (Figure 3A) and CCK assay (Figure 3B). The NV-treated PC12 cells showed increased expression of pAKT and PI3K, which are involved in cell survival (Figure S6) [43]. NV treatment also increased the expression of an anti-apoptotic gene, *Bcl2,* and decreased the expression of an apoptotic gene, Bax (Figure 3C). Next, we investigated the effects of NVs on the phenotype of macrophages in vitro. M1 macrophages are known to be accumulated in inflammatory lesion in the early stage of inflammation. We polarized RAW 264.7 cells, a murine macrophage cell line, into M1 state with LPS for 24 h, and treated the cells with NVs. NVs downregulated the LPS-induced expressions of M1 macrophage markers (*Il1b*, *Il6*, and *Nos2*) and upregulated the expressions of M2 macrophage markers (*Arg1*, *Cd206*, and *Il10*), as evaluated by qRT-PCR (Figure 4), indicating that NVs polarized M1 macrophages into M2 macrophages, which is in agreement with immunostaining results of previous studies [44,45]. The transition of inflammatory M1 macrophages into reparative M2 macrophages in the SCI lesion would relieve inflammation, thereby improving spinal cord repair. Next, we evaluated the angiogenic effects of NVs on HUVECs. Both N-NVs and MF-NVs stimulated the capillary formation and migration of HUVECs under hypoxic conditions (Figure 5A,B). The angiogenic effects of N-NVs and MF-NVs on HUVECs were mediated via the activation of ERK and PCNA signaling cascades (Figure S7) [46].

### 2.3. Enhanced Targeting Efficiency of MF-NVs In Vitro and In Vivo

**Figure 6:**
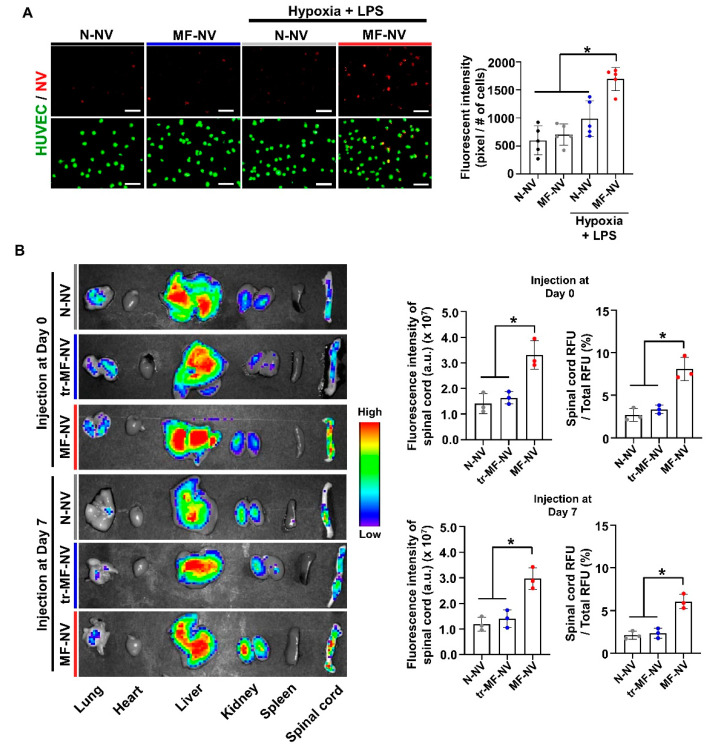
In vitro and in vivo enhanced targeting efficiency of MF-NV. (**A**) Fluorescent images and the quantification data of NV binding to HUVECs in vitro (n = 5 per group). Scale bars, 100 μm. * *p* < 0.05 by using one-way ANOVA followed by post-hoc Bonferroni test. (**B**) Biodistribution of N-NVs, MF-NVs, and tr-MF-NVs in injured spinal cord 24 h after injection 1 h and 7 days post-injury (n = 3 animals per group). Fluorescently labeled NVs were intravenously injected 1 h and 7 days after injury. * *p* < 0.05 by using one-way ANOVA followed by post-hoc Bonferroni test. All values are mean ± SD.

We investigated the potency of MF-NV targeting to ischemic endothelial cells in vitro and injured spinal cord in vivo. Prior to NV treatment, HUVECs underwent hypoxia to mimic spinal cord ischemia. DiI-labeled N-NVs and MF-NVs were added to cultures of DiO-labeled HUVECs for 5 min at 4 °C. The data indicate that MF-NVs exhibited augmented adherence to ischemic endothelial cells (Figure 6A). Next, to determine whether the membrane proteins from macrophages affect the spinal cord targeting efficiency of NVs in vivo, we treated macrophage membranes with trypsin to denature the membrane proteins prior to fuse. Then we fused the trypsin-treated macrophage membranes into MSCs and then produced NVs (tr-MF-NVs) from the fused MSCs. N-NVs, tr-MF-NVs, and MF-NVs were labeled with fluorescent dyes and intravenously injected at 1 h and 7 days post-injury in a mouse compression model of SCI. MF-NV showed and 2.0-fold higher accumulation in the injured spinal cord than N-NV and tr-MF-NV, respectively (Figure 6B). The majority of NVs were accumulated in the liver. However, MF-NVs may not exhibit liver toxicity as previous study described [47]. Taken together, the data suggest that the macrophage membrane components of MF-NVs contribute to the increased targeting efficiency.

### 2.4. Reduced Glial Scar Formation and Improved Function Recovery by MF-NVs In Vivo

**Figure 7:**
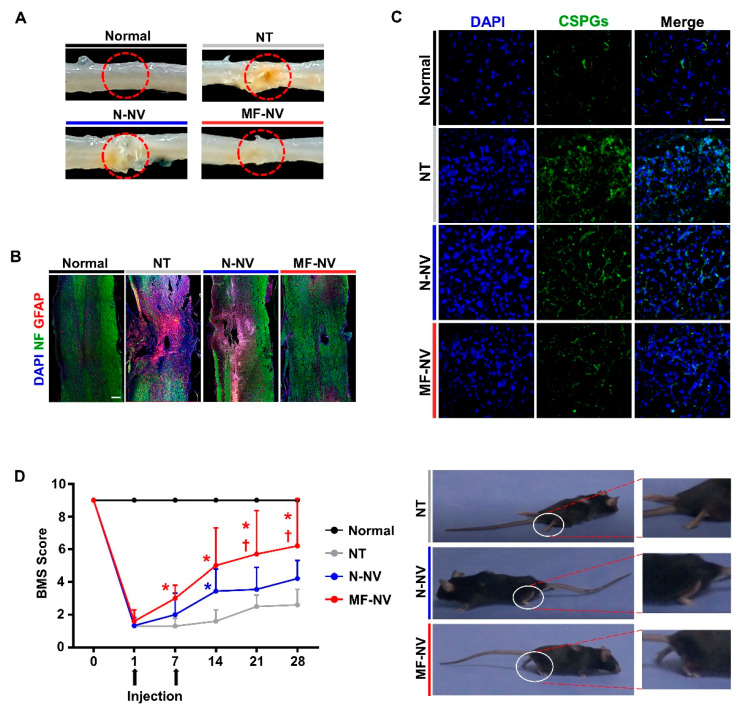
Reduced glial scar formation and improved function recovery by MF-NVs in SCI mice. (**A**) Representative images of spinal cord retrieved 28 days after the NV injection. The dotted red circles indicate the lesion core. (**B**) Representative images of immunohistochemical staining for neuron (NF, green) and astrogliosis (glial fibrillary acidic protein (GFAP), red) in longitudinal sections of spinal cord 28 days post-injury (n = 4 animals per group). Scale bars, 200 μm. (**C**) Representative images of immunohistochemical staining for glial scar-related component (CSPGs, green) at the lesion core 28 days post-injury (n = 4 animals per group). Scale bars, 20 μm. (**D**) Basso mouse scale (BMS) score-based quantitative analysis of time-lapse functional recovery in spinal cord-injured mice and the representative images of injured animals 28 days post-injury (n = 8 animals per group). The white circles indicate the ankle movement. * *p* < 0.05 versus NT and † *p* < 0.05 versus N-NV by using two-way ANOVA followed by post-hoc Bonferroni test. All values are mean ± SD. NT indicates no treatment.

Mice were randomized into four groups: (i) sham group, (ii) the group treated with phosphate-buffered saline (PBS) following static weight compression SCI, (iii) the group treated with N-NV, and (iv) the group treated with MF-NVs. The timeline of the injection and analyses are shown in Figure S8. Therapy was given intravenously 1 h and 7 days post-SCI. Morphological observation showed that SCI-induced lesions were markedly reduced by MF-NVs (Figure 7A). After glial scar formation in SCI, axons cannot regenerate beyond the glial scar [48]. To investigate whether MF-NVs attenuate glial scar formation in standing weight compression mice model of SCI, we performed immunohistochemical (IHC) staining. The SCI-induced lesions were stained for neurofilament (NF), a marker for neuron, and glial fibrillary acidic protein (GFAP), a marker for astroglia, 28 days post-injury (n = 4 mice/group). The no treatment group showed extensive neuronal loss and astrogliosis. MF-NVs showed greater preservation of NF and a significant reduction in GFAP as compared to N-NVs (Figure 7B and Figure S9A,B). The glia scar contains extracellular matrix (ECM) deposited by reactive astrocytes in response to SCI. The scar ECM is rich in chondroitin sulfate proteoglycans (CSPGs), macromolecules that inhibit axonal growth [49]. IHC staining for CSPGs revealed significantly increased expression of CSPGs in the no treatment group day 28 post-injury (Figure 7C and S9C) (n = 4 mice/group). By contrast, MF-NV injection significantly reduced CSPGs expression at the lesion 28 days post-injury compared to the no treatment group. These data indicate that MF-NV could play a crucial role in preventing the deposition of CSPGs.

Basso mouse scale (BMS) score is commonly used to assess functional recovery following SCI in mice (n = 8 mice/group). Before SCI, BMS scores for all mice were 9 points, and the scores on the first postoperative day were 0 (Figure 7D), indicating successful modeling of SCI. After SCI, all mice lost hind limb motor function. Starting from 1 week after injury, the hind limb motor function of the mice gradually recovered till 4 weeks after injury. The scores of the MF-NV injection groups at each time point were significantly higher than those of the other groups. The hind limbs of animals in the MF-NV injection group showed considerable walking ability, whereas animals in the no treatment group exhibited no movement (Supplementary Videos S1–S3). These results demonstrated that locomotion function after static compression SCI was improved by injection of MF-NV.

### 2.5. Enhanced Neuroprotection, Anti-Inflammation, and Angiogenesis by MF-NVs In Vivo

**Figure 8:**
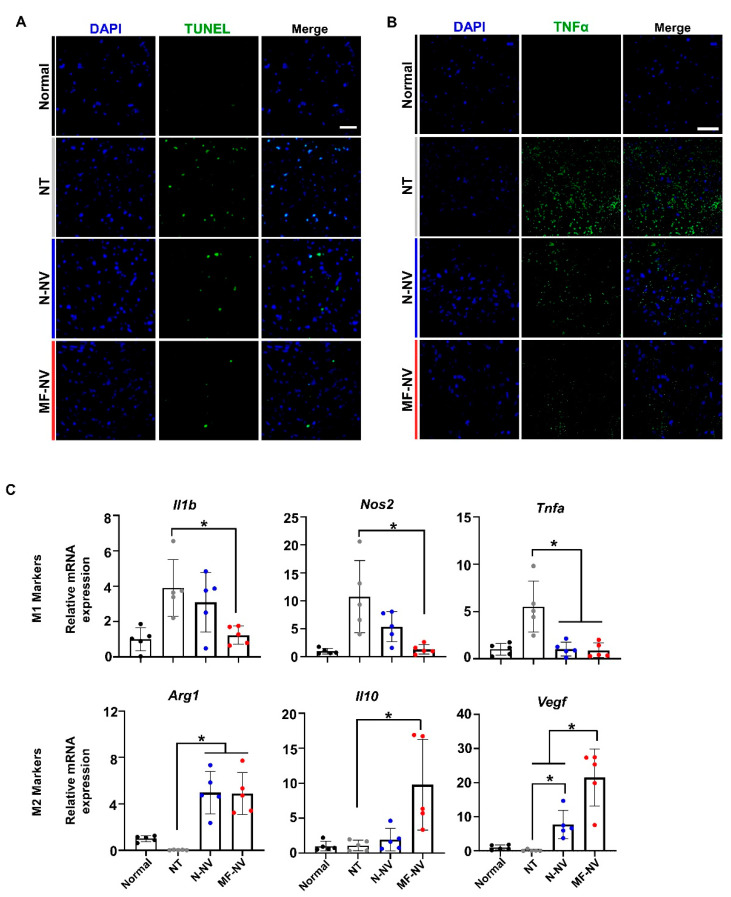

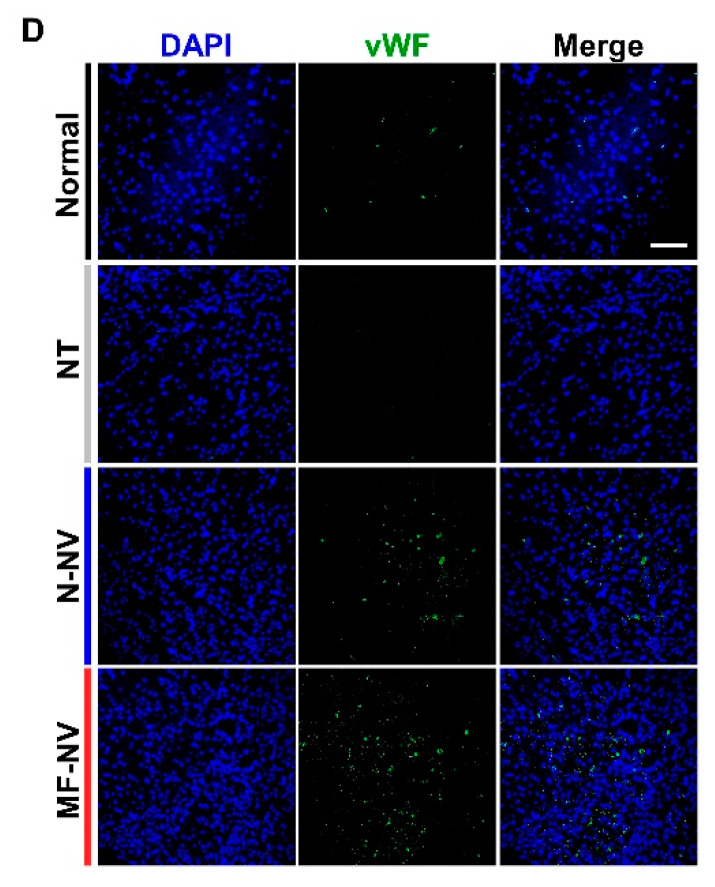
Enhanced neuroprotection, anti-inflammation, and angiogenesis by MF-NVs in the injured spinal cord lesion. (**A**) Representative images of terminal deoxynucleotidyl transferase dUTP nick end labeling (TUNEL) (red)-positive apoptotic cells 7 days post-injury (n = 4 animals per group). Scale bars, 20 μm. (**B**) Representative images of immunohistochemical staining for TNFα 7 days post-injury (n = 4 animals per group). Scale bars, 20 μm. (**C**) Relative mRNA expression levels of M1 macrophage-specific markers (*Il1b*, *Nos2*, and *Tnfa*) and M2 macrophage-specific markers (*Il10*, *Arg1*, and *Vegf*) in the injured spinal cord at 3 days post-injury, as evaluated by qRT-PCR (n = 5 animals per group). * *p* < 0.05 by using one-way ANOVA followed by post-hoc Bonferroni test. All values are mean ± SD. (**D**) Representative images of immunohistochemical staining for blood vessels (vWF, green) at the lesion core 28 days post-injury (n = 4 animals per group). Scale bars, 20μm. NT indicates no treatment.

In vivo therapeutic mechanisms of MF-NVs were investigated. To evaluate the anti-apoptotic and anti-inflammatory effects of MF-NVs in the injured spinal cord, terminal deoxynucleotidyl transferase dUTP nick end labeling (TUNEL) staining assay and immunostaining for tumor necrosis factor α (TNFα) were performed after single intravenous injection 7 days post-SCI (n = 4 mice per group) (Figure 8A,B). The number of TUNEL-positive cells and expression of TNFα in lesion epicenter in the spinal cord were significantly increased after SCI. However, the injection of MF-NV decreased apoptotic cells and expression of TNFα 7 days post-injury (Figure 8A,B and Figure S10A,B).

SCI leads to severe inflammation, microglia and macrophages accumulate at the injured lesion site following trauma to the spinal cord. Indeed, a recent study has described microglial cell and macrophage paradigm associated with SCI. Macrophage activated by microglia can be transformed into M1 (pro-inflammatory) and M2 (anti-inflammatory) [50,51]. The ratio of M1 to M2 macrophages is critical and plays a crucial role in repair following SCI [32,52]. Mostly, M1 macrophages are considered neurotoxic while M2 macrophages are beneficial after SCI. In addition, M2 activation promotes healing activities of microglia. The microglia secrete neurotrophic factors such as brain-derived neurotrophic factor, neurotrophins, and glial cell-derived neurotrophic factor. Therefore, Strategies to increase M2 cell population and decrease M1 cell population in the injured local microenvironment might enhance tissue repair after SCI [53,54]. Therefore, we examined the effects of MF-NVs on M1 and M2 phenotype change in injured spinal cords in mice. The mRNA expression levels of M1 markers—*Il1b*, *Nos2*, and *Tnfa*—decreased and the mRNA expression levels of M2 markers—*Arg1*, *Il10*, and *Vegf*—increased 3 days after injection of MF-NVs (Figure 8C). Taken together, the results suggest that MF-NVs shift the balance from M1 to M2 macrophages at the lesion site and alter the local microenvironment, which is conducive for SCI repair.

Endothelial cells and blood vessels at the injury site in the spinal cord showed degenerative changes within few hours after injury [55]. Significant decreases in the number of vascular endothelial cells were observed subsequently and might cause necrotic or apoptotic cell death. Therefore, we examined whether MF-NV injection could promote angiogenesis after SCI. MF-NV was administrated 1 h and 7 days after SCI by injection. Next, IHC staining for von Willebrand factor (vWF) of the injured spinal cord was performed 28 days post-injury (Figure 8D) (n = 4 mice per group). Surprisingly, the injection of MF-NV significantly increased vWF in the injured spinal cord, whereas the N-NV group did not (Figure 8D and Figure S10C). These data indicate that MF-NVs can enhance neuroprotection and angiogenesis, and attenuate inflammation in SCI mice model, which is attributed to the high targeting efficiency of injected MF-NVs.

## 3. Discussion

In this study, we developed targeting efficiency-enhanced MF-NVs for a cell-free therapy for SCI. Our data suggest that (i) the macrophage membranes can be fused into MSCs and MF-NVs derived from MF-MSCs contained higher levels of targeting molecules than N-NVs derived from MSCs (Figure 2); (ii) MF-NVs exhibited therapeutic efficacy in vitro, which were involved in neuroprotection, anti-inflammation, and angiogenesis (Figure 3, Figure 4 and Figure 5); (iii) MF-NVs showed higher targeting efficiency for hypoxia-conditioned endothelial cells and injured spinal cord in vitro and in vivo, respectively (Figure 6); (iv) in a mouse SCI model, intravenous MF-NV injection facilitated functional recovery of spinal cord via neuroprotection, anti-inflammation, and angiogenesis (Figure 7 and Figure 8).

We confirmed that MF-NVs elicited neuroprotective, anti-inflammatory, and angiogenic effects, which are known as the therapeutic mechanisms of MSC therapies for SCI. We focused on the therapeutic effects of MF-NVs on the cells, however, which molecules in MF-NVs that are involved in the therapeutic mechanisms is still unknown. Further studies will be needed to address this issue. In this study, we injected NVs 1 h after injury and injected NVs again 7 days post-injury. It has been known that spinal cord injury healing process can be divided into several steps depends on time [56]. The inflammation phase occurs within a few days, which is involved in acute inflammation and cellular damages. Subsequently, proliferation and remodeling phases occur, which are involved in angiogenesis, fibrosis (after 3 days post-injury). As a result that therapeutic effects of MF-NVs on spinal cord injury are related to anti-inflammation, neuroprotection, and angiogenesis, we administrated MF-NVs at 1 h and 7 days post-injury. Additional study would be needed to investigate the effects of MF-NVs on inflammatory responses by infiltration of neutrophils and T cells. According to previous studies, the membrane coated nanoparticles from immunocytes including neutrophil and macrophages could target inflammatory lesion and scavenge inflammatory cytokines (e.g., IL1β and TNFα) by the receptors on their membranes [35,36]. This mechanism may make sense in the targeting process and the therapeutic mechanisms of MF-NVs in our study.

Furthermore, the replacement of the cell-line-derived macrophage membranes in the MF-NVs with patient-derived macrophage membranes would increase the possibility of clinical applications of MF-NVs. However, the time period required to culture patient-derived macrophages may limit MF-NV applications to acute SCI patients.

In the summary, we synthetically fabricated MF-NVs for spinal cord repair. MF-NVs exhibited: (i) neuroprotective effects by reducing apoptosis of neuronal cells; (ii) angiogenic effects by increasing migration and tube formation of endothelial cells; and (iii) anti-inflammatory effects by polarizing M1 to M2 macrophages in vitro, which are closely associated with the mechanism of spinal cord repair. Importantly, incorporation of the macrophage plasma membrane into MSC-derived NVs significantly improved the injured spinal cord-targeting efficiency of MSC-derived NVs and potentiated the therapeutic efficacy of MSC-derived NVs for SCI.

## 4. Materials and Methods

### 4.1. Cell Culture

Human umbilical cord blood-derived MSCs were purchased (CEFO, Seoul, South Korea) and cultured according to the manufacturer’s instructions. MSCs were cultured in Dulbecco’s modified Eagle’s medium low glucose (Gibco BRL, Gaithersburg, MD, USA) supplemented with 10% (v/v) fetal bovine serum (FBS) (Gibco BRL) and 1% (v/v) penicillin/streptomycin (Gibco BRL) and were used for the experiments at passage under six. RAW 264.7 cell line was purchased from Korean Cell Line Bank (Seoul, South Korea) and cultured according to the manufacturer’s instructions. RAW 264.7 cells were cultured in Dulbecco’s modified Eagle’s medium high glucose (Gibco BRL) supplemented with 10% (v/v) FBS and 1% (v/v) penicillin/streptomycin. PC12 cells were purchased from Paragon Biotech (Baltimore, MD, USA) and cultured in RPMI 1640 (Gibco BRL) supplemented with 7.5% (v/v) FBS, 7.5% (v/v) horse serum (Gibco BRL), and 1% (v/v) penicillin/streptomycin. HUVECs were purchased from Lonza (Walkersville, MD, USA) and cultured in Endothelial Cell Growth Media-2 (Lonza) on tissue culture plates. HUVECs at a passage under seven were used for the experiments.

### 4.2. Isolation of Macrophage Membranes

The plasma membrane of RAW 264.7 cell line was isolated as previously described [57]. Briefly, RAW 264.7 cell line was suspended in a hypotonic buffer, followed by sonication for 5 min and then centrifuged at 10,000× *g* for 20 min. The pellet was discarded and the supernatant was extruded through 400 nm polycarbonate membrane filter. Then the sample was centrifuged at 25,000× *g* for 30 min. Approximately 1 × 10^9^ membrane particles were produced from 1 × 10^7^ cells. The membranes were stored at −80 °C before use.

### 4.3. Generation and Characterization of MF-MSCs

Macrophage membrane was fused to MSCs using polyethylene glycol (PEG) as previously described [58]. Briefly, 1 × 10^6^ MSCs and 1 × 10^10^ membrane particles were mixed in 100 μL of PEG (MW 1500) for 5 min. The mixed sample was then diluted by 5 mL of phosphate-buffered saline (PBS) and followed by centrifugation at 500× *g* for 5 min to separate free macrophage membranes. MSCs and MF-MSCs were suspended in PBS and were photographed using a fluorescence microscope (Olympus, Tokyo, Japan). To identify the surface marker of MSCs and MF-MSCs, Western blot analysis and flow cytometry were used. To produce trypsin-treated macrophage membrane-fused MSCs (tr-MF-MSCs), macrophage membranes were treated with 2.14 M trypsin for 30 min at 37 °C prior to fusion. The process of fusion was the same with that of MF-MSCs, as described earlier.

### 4.4. Generation and Characterization of MF-NVs

NVs were prepared from MSCs, MF-MSCs, and tr-MF-MSCs using a modification of a previous method [30]. Cells were suspended in PBS at 1 × 10^6^ cells/mL and extruded 4–5 times through a series of 10 μm, 5 μm, and 400 nm-sized pore polycarbonate membrane filters (Whatman Inc., Clifton, NJ, USA) using a mini extruder (Avanti Polar Lipids). An iodixanol gradient solution (Axis-Shield PoC AS, Oslo, Norway) was used to isolate NVs from the mixture of cellular debris and free proteins. One mL of 50% iodixanol, 2 mL of 10% iodixanol, and 7 mL of the samples were placed sequentially onto the bottom of a tube, and the tube was centrifuged at 100,000× *g* for 2 h at 4 °C. After centrifugation, the second layer of three layers was collected and an additional centrifugation at 100,000× *g* for 2 h was performed to collect NVs. The NV pellets in the tube were resuspended in PBS and filtered with 0.45 μm syringe filter prior to use. The protein quantities of NVs were determined using Bradford assay as previously described [31]. Approximately 100–150 μg of NVs were produced from 1 × 10^6^ cells. Each NV sample prepared from each batch of cell culture was used for each replicate (n = 3–5) in the in vitro and in vivo experiments. The size distributions of N-NVs and MF-NVs were determined using Nanoparticle Tracking Analysis using Nanosight (LM10, Malvern, UK). To visualize the morphology of N-NVs and MF-NVs, TEM analysis was used. To identify the surface markers of N-NVs and MF-NVs, Western blot analysis was used.

### 4.5. Western Blot Analysis

Western blot analysis was used to evaluate the expression levels of protein in cells and NVs. Cell, NV, and membrane samples were lysed using SDS sample buffer (6.25 × 10^−3^ M Tris-HCl pH 6.8, 2% (w/v) of SDS, 10% (v/v) of glycerol, 50 × 10^−3^ M dithiothreitol, and 0.1% (w/v) bromophenol blue). Proteins in the sample buffer were separated by 10% SDS polyacrylamide gel electrophoresis. Then we transferred the gels to an Immobilon-P membrane (Millipore Corp., Bedford, MA, USA). For protein detection, primary antibodies against CD68, integrin α4, integrin β1, CD9, ERK1/2, pERK1/2, PCNA, β-actin (these antibodies were purchased from Abcam, Cambridge, UK), AKT, pAKT, and PI3K (these antibodies were purchased from Cell Signaling Technology, MA, USA) were incubated with membrane overnight at 4 °C. The membranes then were washed and incubated with secondary antibodies for 1 h at room temperature. The blots were developed by chemiluminescence reagent (LumiGLO, KPL Europe, Guildford, UK). Quantification of the Western blots was evaluated using the ImageJ software version 1.52r.

### 4.6. Flow Cytometry

RAW 264.7 cells, MSCs, and MF-MSCs were detached using a cell scraper and Trypsin-EDTA and then made into single-cell suspensions. Cells were stained with antibodies against CD90 and F4/80 which are conjugated with FITC and Alexa 647 (Biolegend, San Diego, CA, USA), respectively. Then cells were analyzed by FACS cantoII (BD Bioscience, San Jose, CA, USA).

### 4.7. In Vitro Therapeutic Effects of MF-NVs

To generate hypoxic culture conditions for PC12 cells and HUVECs, the cells were cultured in the media containing 500 μM of H_2_O_2_ and lipopolysaccharide (LPS) (100 ng/mL, Sigma) in a hypoxic incubator (MCO-18M, Sanyo, Japan) containing 1% O_2_ at 37 °C for 24 h. To polarize macrophages into M1 phenotype, RAW 264.7 cells were cultured in the presence of LPS (100 ng/mL) for 24 h in normoxia. To examine the therapeutic mechanisms of N-NVs and MF-NVs in vitro, the cells were treated with N-NVs and MF-NVs (20 μg/mL) for 24 h under hypoxic and polarization culture conditions. Twenty four hours after the NV treatment, the medium was changed and the cells were analyzed after further 24 h. The neuroprotective effects of NVs on PC12 cells were evaluated by LIVE/DEAD staining, CCK assay, and quantitative PCR. For LIVE/DEAD staining, fluorescein diacetate (FDA) and ethidium bromide (EB) solution were used. A total of 1 mL of FDA (5 μg/mL, Sigma) and EB (10 μg/mL, Sigma) mixed solution was added to each well of 6 well culture plates and incubated 5 min at 37 °C. The samples were then photographed with a fluorescence microscope (Olympus). For CCK assay, 100 μL/mL of EZ-cytox (Daeillab service, Changwon, South Korea) was added to 24 well culture plates and incubated for 2 h at 37 °C. The absorbance of the solution was measured at 450 nm using a spectrophotometer. The polarization of macrophages was determined by quantitative PCR. The angiogenic effects of NVs on HUVECs were evaluated by capillary tube formation assay and cell migration assay. For capillary tube formation assay, culture plates were coated with Geltrex (Invitrogen) for 30 min at 37 °C to promote gelation. HUVECs were cultured under hypoxic condition (1% O_2_) with 500 μM of H_2_O_2_ for 24 h followed by NV treatment (6 h, 20 μg/mL). Then HUVECs were detached and plated on the Geltrex (5 × 10^4^ cells/cm^2^) for 6 h at 37 °C. Capillary tubes were observed using a light microscope. Five random fields were measured for the quantitative analysis. For cell migration assay, HUVECs were seeded and grown until confluence. HUVECs underwent hypoxic condition (1% O_2_) with 500 μM of H_2_O_2_ for 24 h followed by NV treatment (6 h, 20 μg/mL). Then we created a linear gap by scratching the surface of the wells using a sterile yellow tip. The cells were washed three times with PBS to remove the detached cells. After 12 h, five random fields were photographed. For quantitative evaluation, the following equation was used: [(initial cell-free area–cell-free area at 12 h) / initial cell-free area] × 100%. The scratched area was evaluated using the Image J software.

### 4.8. Quantitative PCR

The expression levels of mRNA were evaluated using qRT-PCR. Cell (n = 4) and tissue samples (n = 5) were lysed using 1 mL of the TRIzol reagent (Invitrogen, Carlsbad, CA, USA). To evaluate mRNA expressions in spinal cord, the lesions of spinal cords were collected and homogenized. We extracted total RNA using 200 μL of chloroform. The mixture was centrifuged at 10,000× *g* for 10 min at 4 °C. The supernatant was collected, mixed with 80% (v/v) isopropanol, and centrifuged at 10,000× *g* for 10 min at 4 °C. The RNA pellets were then washed with 75% (v/v) ethanol and dissolved in RNase-free water. Complementary DNA from RNA were evaluated using the StepOnePlus real-time PCR system (Applied Biosystems, Foster city, CA, USA). Glyceraldehyde 3-phosphate dehydrogenase served as the internal control. Primer sequences that were used for analysis are described in Tables S1 and S2.

### 4.9. In Vitro NV Binding Assay

HUVECs were labeled with a fluorescent dye, DiO, for 2 h prior to exposure to hypoxia. Then HUVECs were cultured under hypoxic condition (1% O_2_) in the medium containing 500 μM of H_2_O_2_ for 24 h. The cells were then treated with DiI-labeled N-NVs and MF-NVs in PBS (20 μg/mL) for 5 min at 4 °C to prevent endocytosis of N-NVs and MF-NVs. After NV binding to HUVECs, the cells were washed with PBS 3 times and photographed with a fluorescence microscope (Olympus). Total red fluorescent intensity was measured using Image J software. The fluorescent intensity was divided by HUVEC numbers for the quantification.

### 4.10. SCI Model and Treatment

Female 6- to 8-week-old C57BL/6 mice (20–25 g, Koatec Inc, Korea) were deeply anesthetized with an intraperitoneally administered mixture of Zoletil^®^ (50 mg/kg, Virbac Laboratories, France) and Rompun^®^ (10 mg/kg, Bayer, Korea). To induce standing weight compression model of SCI, laminectomy was made at the ninth thoracic vertebral levels (T9) and a stainless steel impounder (weight: 20 g) was loaded to the T10 spinal cord for 30 sec after laminectomy. Following compression lesion, animals were placed on a heating pad to maintain body temperature, and then 0.5 mL of 0.9% sterile saline injected subcutaneously. Manual bladder expression of urine was performed twice daily until a reflex bladder was established. All animal procedures were performed according to the approved protocol by the Institutional Animal Care and Use Committee (IACUC) of CHA University (IACUC180099, May 29, 2018). The mice were randomly assigned into four groups; (i) sham group (laminectomy alone), (ii) no treatment group: injury group treated with 100 μL PBS injection, (iii) N-NV treatment group: injury group treated with 25 μg of N-NV in 100 μL PBS, and (iv) MF-NV treatment group: injury group treated with 25 μg of MF-NV in 100 μL PBS. Therapeutic materials were intravenously injected to tail vein 1 h and 7 days post-SCI. A total of 23 mice per group were used for short- and long-term study (Figure S8). We used 6 mice animals per group for assessing biodistribution of therapeutic materials 24 h post-injection at 1 h (n = 3 animals per group) and day 7 (n = 3 animals per group) post-SCI (Figure 6). For RT-PCR to investigate the expression levels of M1- and M2-related genes, we used 5 mice animals per group 3 days post-SCI (Figure 8). For TUNNEL assay and immunostaining for TNFα (n = 4 animals per group), we sacrificed 4 mice per group before the second injection of therapeutic materials 7 days post-SCI (Figure 8). Eight mice were intravenously injected with each therapeutic material 1 h and 7 days post-SCI and BMS score was evaluated every week for 28 days. Twenty-eight days post-SCI, immunostaining for CSPG, vWF, NF, and GFAP was performed (Figure 7 and Figure 8).

### 4.11. Ex Vivo Biodistribution of NVs

To evaluate the biodistribution of N-NVs, tr-MF-NVs, and MF-NVs, the plasma membranes of NVs were labeled with Vivotrack 680 (PerkinElmer, MA, USA) for 2 h prior to injection. The fluorescent signal of NVs was detected using an in vivo imaging system (IVIS spectrum, PerkinElmer) 1 day after the intravenous injection into SCI mice 1 h and 7 days post-injury. The fluorescent images of organs (lung, heart, liver, kidney, spleen, and spinal cord) were obtained by removing background with no treatment group. Then the fluorescent intensity in the spinal cord was quantified. The relative fluorescent units were quantified by the following equation: [(fluorescent intensity in spinal cord/total fluorescent intensity of all organs) × 100%]. The fluorescent intensities of the spinal cord and other organs were measured using the Living Image version 3.1 software.

### 4.12. Immunohistochemical Assessment In Vivo

Mice were perfused with 4% paraformaldehyde for tissue fixation. Spinal cord tissues were paraffin-embedded and sectioned at 5–10μm thickness. Transverse and longitudinal sections were obtained by a microtome (Leica RM2255, Leica Instruments, Nußloch, Germany). The sections were deparaffinized and rehydrated. By heating at 60 °C followed by washing in xylene and rehydration through a graded series of ethanol (50–100% at 4 °C) and double-distilled water. Tissue sections were incubated in 3% H_2_O_2_ for 10 min, followed by incubation in Pepsin for 10 min. Then, the tissue sections were stained with primary antibodies against vWF, GFAP, NF, CSPGs, and tumor necrosis factor alpha (TNFα). Alexa 488 or Alexa 546-conjugated secondary antibodies (Invitrogen) were applied to primary antibody-incubated samples with different color combinations. The fluorescent intensity of each protein in DAPI-positive cells was quantified using ImageJ software (n = 4 animals per group).

### 4.13. In Vivo Apoptosis Assessment

TUNEL assay was performed to detect apoptotic cells with the in situ cell death detection kit (Roche). Briefly, fixed spinal cord sections were permeabilized and incubated with fluorescein TUNEL reaction mixture containing for 60 min at 37°C in dark. After washing with PBS, nuclei were stained with DAPI (300 ng/mL in PBS, Sigma-Aldrich). To quantify TUNEL-/DAPI-positive cells, photomicrographs were taken using confocal microscope (Zeiss LSM 880, Zeiss, Oberkochen, Germany) with a 20× objective.

### 4.14. Behavior Evaluation

Behavioral analysis was conducted by two observers, blinded to the treatment identity. Coordinated motor function was evaluated with the BMS locomotor rating scale (scored on a 9-point scale) [59]. To evaluate functional recovery, ten mice were randomly selected from each group at 7, 14, 21, and 28 days after surgery. The mice were observed continuously from 1 min.

### 4.15. Statistical Analysis

All quantitative data are expressed as the mean ± standard deviation. In case of the comparison between two groups, a two-tailed student’s t-test was used. A one-way analysis of variance (ANOVA) followed by the Bonferroni test was used for other statistical analyses. For BMS score analysis was analyzed using two-way ANOVA followed by the Bonferroni test. A difference with a *p* value less than 0.05 was considered as statistically significant. All statistical analyses were performed using Prism version 8.0 (GraphPad Software Inc.).

## Supplementary Materials

Supplementary materials can be found at https://www.mdpi.com/1422-0067/21/11/4185/s1.

## Abbreviations

SCI	Spinal cord injury
MSC	Mesenchymal stem cell
NV	Exosome-mimetic nanovesicle
VLA4	Very late antigen 4
FBS	Fetal bovine serum
PBS	Phosphate buffered saline
LPS	Lipopolysaccharide
ERK	Extracellular signal-regulated kinases
AKT	Serine/threonine-specific protein kinase
PI3K	Phosphatidylinositol 3-kinases
PCNA	Proliferating cell nuclear antigen
HUVEC	Human umbilical vein endothelial cell
qRT-PCR	Quantitative real-time polymerase chain reaction
vWF	von Willebrand factor
Arg1	Arginase 1
NF	Neurofilament
GFAP	Glial fibrillary acidic protein
CSPG	chondroitin sulfate proteoglycans
TUNEL	Terminal deoxynucleotidyl transferase dUTP nick end labeling
TNFα	Tumor necrosis factor alpha
BMS	Basso mouse scale
PEG	Polyethylene glycol
TEM	Transmission electron microscopy
FACS	Fluorescence-activated cell sorting
IL	Interleukin
Nos2	Nitric oxide synthase 2
IHC	Immunohistochemistry
Vegf	Vascular endothelial growth factor
ECM	Extracellular matrix
