# Supplementary material for: Targeted Delivery of Mesenchymal Stem Cell-Derived Nanovesicles for Spinal Cord Injury Treatment
Source: Int J Mol Sci. 2020 Jun 11;21(11):4185. doi: 10.3390/ijms21114185 (PMC7312698; doi:10.3390/ijms21114185)
Supplement: Supplementary file 1 [file ijms-21-04185-s001.zip › ijms-818756-supplementary.pdf]

Supporting Information

Targeted Delivery of Mesenchymal Stem Cell-Derived Nanovesicles for Spinal Cord Injury Treatment

Ju-Ro Lee, Jaewon Kyung, Hemant Kumar, Sung Pil Kwon, Seuk Young Song, In-Bo Han and Byung-Soo Kim**

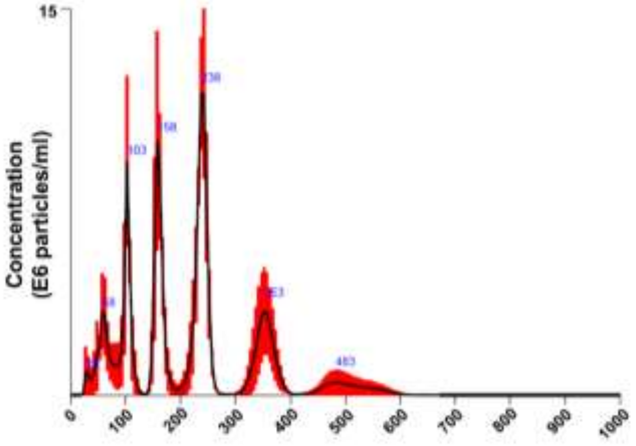

Figure S1. Characterization of MΦ membrane. Size distribution of MΦ membrane, as evaluated by nanoparticle tracking analysis.

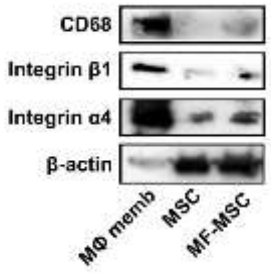

Figure S2. Representative images of western blots of surface markers of macrophage membrane, MSC, and MF-MSC in Figure 2B (n = 3 per samples, 20 μg of total proteins).

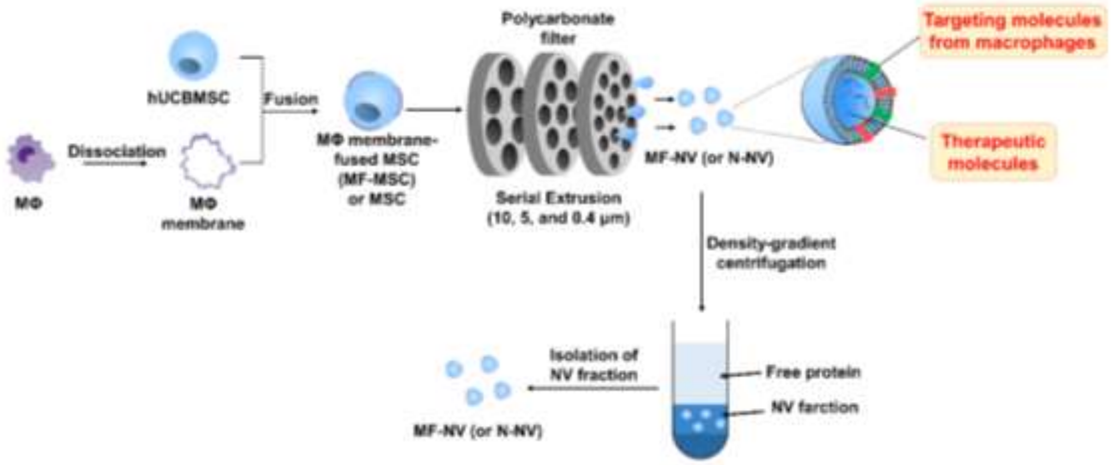

Figure S3. Preparation of MF-NVs. Schematic diagram of the preparation of MF-NVs.

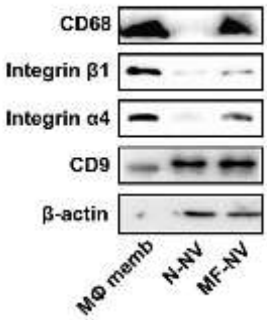

Figure S4. Representative images of western blots of surface marker of macrophage membrane, N-NV, and MF-NV in Figure 2F (n = 3 per samples, 20 μg of total proteins).

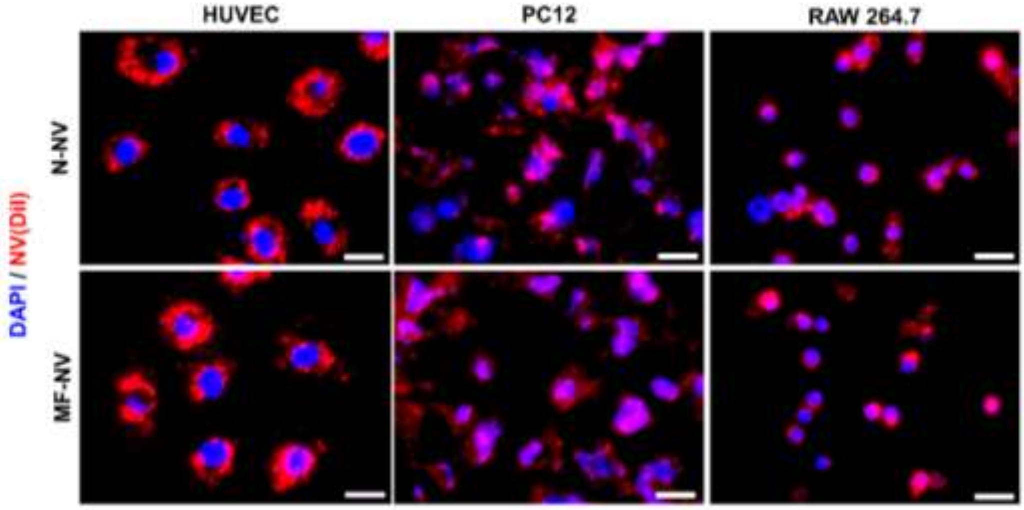

Figure S5. Cellular uptake of NVs. Representative images showing in vitro uptake of DiI-labeled N-NVs and MF-NVs by HUVECs, PC12 cells, and RAW 264.7 cells 24 hours after the treatment. Scale bars, 50 μm.

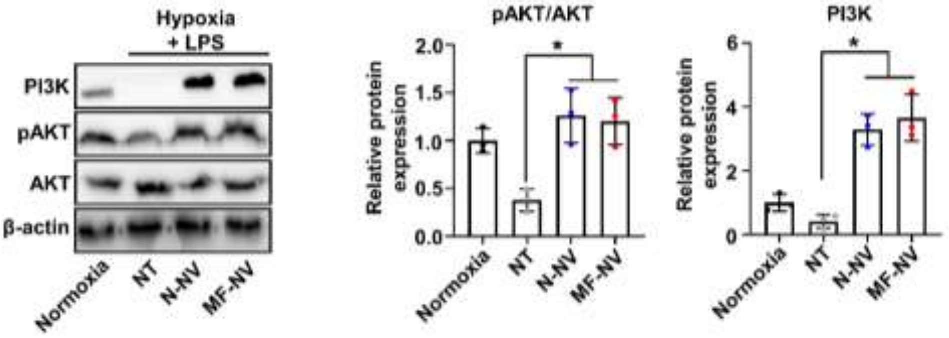

Figure S6. Intracellular signaling cascades of PC12 cells after treatment. Representative images and the quantification data of western blots of PC12 cells for AKT, pAKT, and PI3K after treatment. *$p < 0.05$ by using one-way ANOVA followed by post-hoc Bonferroni test. All data were normalized to normoxia. All values are mean ± SD. NT indicates no treatment.

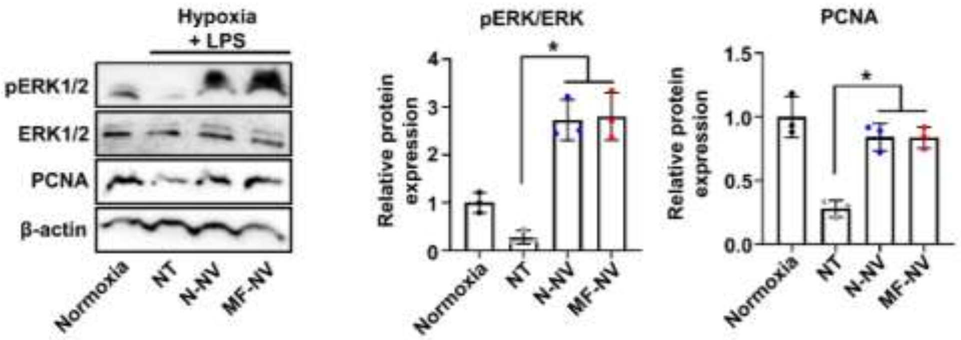

Figure S7. Intracellular signaling cascades of HUVECs after treatment. Representative images and the quantification data of western blots of HUVECs for PCNA, ERK 1/2, and pERK 1/2 after treatment. *$p < 0.05$ by using one-way ANOVA followed by post-hoc Bonferroni test. All data were normalized to normoxia. All values are mean ± SD. NT indicates no treatment.

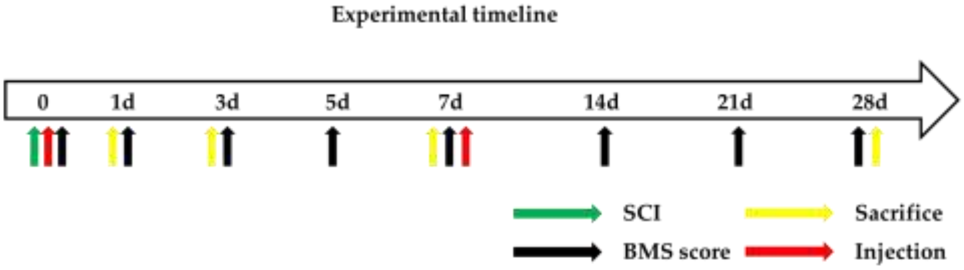

	Analysis & time point			
1st injection	Bio-distribution of therapeutic materials	RT-PCR	TUNNEL assay	immunostaining for TNF-α
Number of animals	n=6/group	n=5/group	n=4/group	
Post-injection	1day/7day	3day	7day	
2nd injection	BMS score		immunostaining for CSPG, vWF, NF, and GFAP	
Number of animals	n=8/group			
Post-injection	28day			

Figure S8. In vivo Experimental Design and timeline.

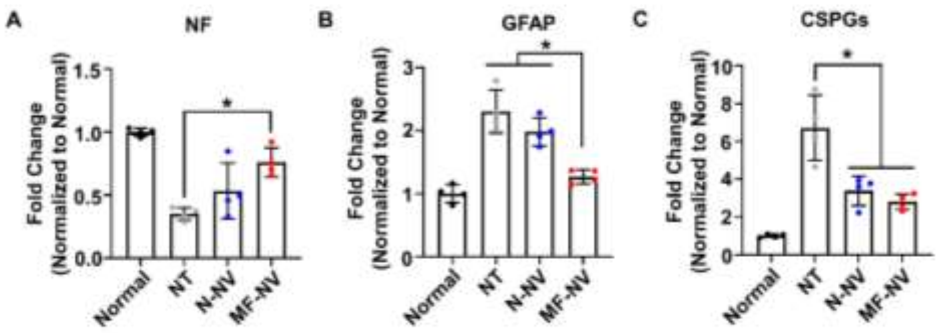

Figure S9. The quantification data of IHC images in Figure 7. IHC images of (**A**) NF, (**B**) GFAP, and (**C**) CSPGs were quantified, as evaluated by ImageJ software (n = 4 animals per group). The data of fluorescent intensity per area were normalized to normal group. **p* < 0.05 by using one-way ANOVA followed by post-hoc Bonferroni test. All values are mean ± SD. NT indicates no treatment.

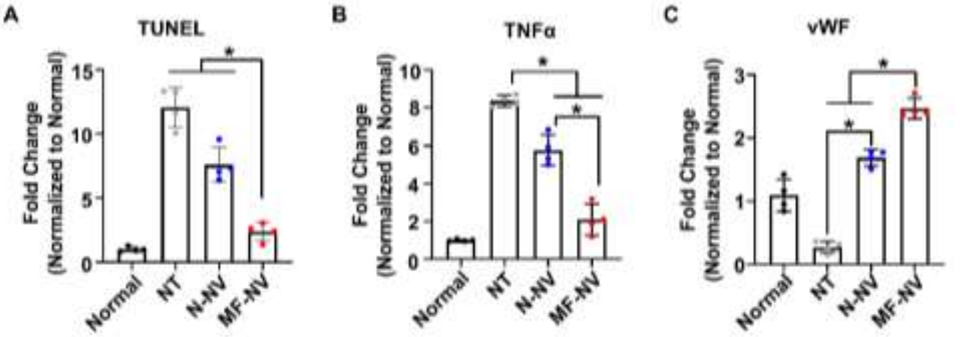

Figure S10. The quantification data of IHC images in Figure 8. IHC images of (**A**) TUNEL, (**B**) TNFα, and (**C**) vWF were quantified, as evaluated by ImageJ software (n = 4 animals per group). The data of fluorescent intensity per area were normalized to normal group. **P* < 0.05 by using one-way ANOVA followed by post-hoc Bonferroni test. All values are mean ± SD. NT indicates no treatment.

Table S1. List of PCR primers of mouse-specific genes. *Il1b,* interleukin 1 beta; *Il6* interleukin 6; *Nos2,* nitric oxide synthase 2; *Arg1,* arginase 1; *Il10,* interleukin 10; *Tnfa,* tumor necrosis factor alpha; *Vegf,* vascular endothelial growth factor.

**Gene**	**Primers**	
	**Forward (5′-3′)**	**Reverse (5′-3′)**
Gapdh	GCATCTTCTTGTGCAGTGCC	GGTAACCAGGCGTCCGATAC
Il1b	ATCAGGACAGCCCAGGTCAA	GCCACCTTTTGACAGTGATGAG
Il6	GAGGATACCACTCCCAACAGACC	AAGTGCATCATCGTTGTTCATACA
Nos2	TCACCTTCGAGGGCAGCCGA	TCCGTGGCAAAGCGAGCCAG
Arg1	GATTATCGGAGCGCCTTTCT	CCACACTGACTCTTCCATTCTT
Cd206	CTGCAGATGGGTGGGTTATT	GGCATTGATGCTGCTGTTATG
Il10	ACTGGCATGAGGATCAGCAG	CTCCTTGATTTCTGGGCCAT
Tnfa	ACCCTCACACTCACAAACCA	GCAGCCTTGTCCCTTGAAGA
Vegf	CAGGCTGCTCTAACGATGAA	CAGGAATCCCAGAAACAACC

Table S2. List of PCR primers of rat-specific genes.

**Gene**	**Primers**	
	**Forward (5′-3′)**	**Reverse (5′-3′)**
Gapdh	GCATCTTCTTGTGCAGTGCC	GGTAACCAGGCGTCCGATAC
Bax	ATGAATGGGGAACGGGGAAA	GAGGTTTATTGGCACCTCCCC
Bcl2	CTGGTGGACAACATCGCTCT	CATCCCAGCCTCCGTTATCC

Supplementary video 1. Representative video of animals in the no treatment group 28 days post-injury.

Supplementary video 2. Representative video of animals in the N-NV group 28 days post-injury.

Supplementary video 3. Representative video of animals in the MF-NV group 28 days post-injury.
